# Transformers in EEG Analysis: A Review of Architectures and Applications in Motor Imagery, Seizure, and Emotion Classification

**DOI:** 10.3390/s25051293

**Published:** 2025-02-20

**Authors:** Elnaz Vafaei, Mohammad Hosseini

**Affiliations:** 1 Department of Psychology, Northeastern University, Boston, MA 02115, USA; 2 Department of Biomedical Engineering, Science and Research Branch, Islamic Azad University, Tehran 1477893855, Iran

**Keywords:** transformers, vision transformer, graph attention transformer, electroencephalography (EEG), brain–computer interface (BCI), motor imagery classification, emotion recognition, seizure detection

## Abstract

Transformers have rapidly influenced research across various domains. With their superior capability to encode long sequences, they have demonstrated exceptional performance, outperforming existing machine learning methods. There has been a rapid increase in the development of transformer-based models for EEG analysis. The high volumes of recently published papers highlight the need for further studies exploring transformer architectures, key components, and models employed particularly in EEG studies. This paper aims to explore four major transformer architectures: Time Series Transformer, Vision Transformer, Graph Attention Transformer, and hybrid models, along with their variants in recent EEG analysis. We categorize transformer-based EEG studies according to the most frequent applications in motor imagery classification, emotion recognition, and seizure detection. This paper also highlights the challenges of applying transformers to EEG datasets and reviews data augmentation and transfer learning as potential solutions explored in recent years. Finally, we provide a summarized comparison of the most recent reported results. We hope this paper serves as a roadmap for researchers interested in employing transformer architectures in EEG analysis.

## 1. Introduction

Electroencephalogram (EEG) is a non-invasive and cost-effective method for recording and monitoring electrical brain activity, captured by electrodes placed on a scalp. EEG exhibits neural oscillations and brain dynamics to process real-time brain states with high temporal resolution, enabling the detection and investigation of brain disorders. EEG has attracted significant attention for various applications and research fields, including brain–computer interfaces (BCIs) [1], emotion classification [2], seizure detection [3], monitoring sleep stages [4], and the exploration of diverse mental disorders and cognitive functions [5]. EEG signals are inherently dynamic and stochastic, with short- and long-range dependencies, influenced by individual characteristics and environmental factors [6]. The biological complexities, artifacts from physiological and non-physiological sources, motion artifacts, and environmental noise pose significant challenges to EEG interpretability.

Over the past few decades, numerous studies have been conducted to develop advanced analytical methods, including signal processing techniques and machine learning algorithms, to enhance EEG analysis and uncover the deeper mechanisms of brain function. Traditional machine learning models, such as Support Vector Machines (SVMs) [7], random forests [8], autoencoders [9], and decision trees [10], have been widely used in EEG analysis for classification, clustering, and regression tasks [11]. While these models offer simple implementation and robust performance for small amounts of data, they have limitations and major difficulties in handling the complex and dynamic nature of EEG data.

Additionally, conventional models often require extensive feature extraction and, in many cases, fail to capture the underlying patterns of EEG signals. Deep learning models have been introduced to address these limitations. Among deep learning models, Convolutional Neural Networks (CNNs) and Long Short-Term Memory (LSTM) networks have demonstrated strong performance in EEG analysis [12,13,14]. CNNs are capable of hierarchically extracting complex patterns from EEG data, and LSTMs have demonstrated considerable performance in handling sequential data [15]. Nevertheless, CNNs encounter challenges in effectively capturing long-range dependencies [16]. Furthermore, the sequential nature of LSTM results in a slower training process and faces challenges in handling long-term dependencies, primarily due to the vanishing gradient problem [17].

Transformers were initially introduced in natural language processing (NLP) [18]. NLP models like BERT and GPT-3 emerged based on the transformer architecture and have rapidly evolved into more advanced models with hundreds of millions of parameters [19,20]. These models are continuously improving and extending into various aspects of our lives. They have revolutionized knowledge expansion and, in some cases, have even become the primary search engines. However, they struggle with small-sized datasets and require extensive fine-tuning on large datasets to achieve state-of-the-art performance in NLP applications. For instance, GPT-3 is an autoregressive language model with 175 billion parameters and has demonstrated strong performance in many NLP tasks, such as translation and question answering. More advanced models go even further, such as GPT-4, a multimodal model that accepts both image and text inputs and produces text output, aiming to exhibit human-level performance in some areas [21].

Transformers have rapidly revolutionized machine learning in many fields, including computer vision [22], graph analysis [23], and time series analysis [24]. Before the expansion of transformer models, CNNs achieved the highest performance in vision classification tasks. Vision Transformers [22] demonstrated that convolutional layers are not necessarily required, as the attention mechanism of transformers can be directly applied to a series of image patches, outperforming CNNs in classification tasks. Very quickly, more advanced vision models, such as the Swin Transformer [25], emerged as general-purpose backbones for computer vision, addressing the large variations in the scale of visual entities and the high resolution of image pixels. Like their original transformer counterparts, they struggle with limited dataset sizes and contain hundreds of millions of parameters. The advancement of Vision Transformers is rapid, with new models being released frequently. Furthermore, they have expanded beyond image classification to image generation and perception. Graph Attention Transformers are innovative models that are rapidly expanding and apply attention to graph-structured data. Based on the efficiency of attention on graphs, they have a few million parameters and exhibit better generalization compared to transformers in the NLP and vision fields [23]. However, they struggle with large datasets due to the lack of pre-trained models and limited transferability to other domains.

**Figure 1:**
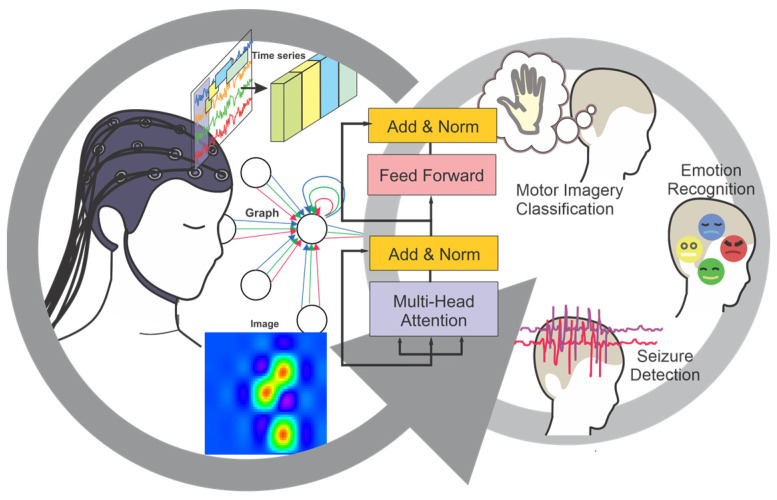
Applications of transformers in EEG analysis.

Transformers can capture long-range sequential data and efficiently handle underlying relationships within the data [26]. Their parallel processing architecture enables the effective handling of large datasets, making them highly computationally efficient. With the success of transformer models, EEG studies have shown a growing interest in applying these models, and the highly promising results have led to a sharp increase in research activity. Successful transformer-based EEG studies have demonstrated superior performance compared to other deep learning models. EEG is an informative multi-channel signal that can be represented in the form of time series [27], images [28], graphs [29], or sequences of features [30]. The rich nature of EEG provides an opportunity for employing various transformer structures, expanding the scope of EEG analysis studies. There is little literature investigating transformer architectures and their variants in EEG analysis. For instance, the literature [31] provides a review of transformer applications in neuroscience, with a focus on brain imaging analysis such as PET and MRI. Although this review explores EEG analysis to a lesser extent, the authors provide a clear understanding of the impact of transformers in neurology and psychiatry as well. Despite the growing interest and the increasing number of published studies, there remains a lack of a comprehensive survey that categorizes fundamental transformer types, explains their structures, and examines their adaptations for EEG analysis. This review aims to address this gap by presenting a framework for transformer architecture variants and their adaptations in EEG analysis. An overview of the current survey is presented in Figure 1. The objective of this review is to provide a basic outline of transformer architectures and their applications, serving as a practical guide for gaining insights into designing EEG studies.

This paper is structured as follows. Section 2 provides a brief overview of vanilla transformer architectures and expounds on their key components. Subsequently, Section 3 reviews four major types of transformers: Time Series, Vision, Graph Attention, and hybrid transformers, highlighting how these structures have been adapted and applied in EEG research. Section 4 presents transformer applications in EEG analysis and categorizes them, based on frequent studies, into four main areas: motor imagery classification, emotion recognition, seizure detection, and other less frequent applications. This section also discusses the recent research developments in these domains, with summarizing models, applications, and performance from the past year. Section 5 addresses the challenges encountered when using transformers in EEG analysis and explores strategies to overcome them, offering insights for future directions. Finally, in Section 6, we discuss and outline the review paper, providing guidelines for researchers.

## 2. Vanilla Transformer

**Figure 2:**
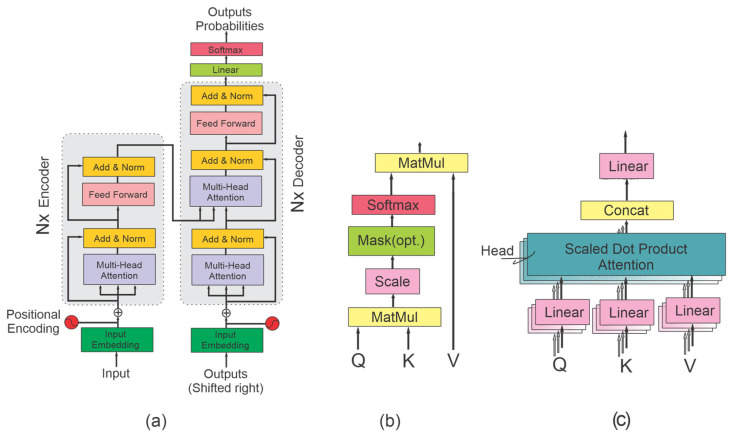
Vanilla transformer proposed by [18]. (**a**) Transformer composed of encoder and decoder; (**b**) attention mechanism; (**c**) multi-head attention.

The vanilla transformer model, introduced by Vaswani et al. in the 2017 paper “Attention Is All You Need”, was originally designed for NLP applications [18]. It is a sequence-to-sequence architecture, consisting of an encoder and a decoder, both composed of stacks of identical layers designed to capture sequential dependencies in input data. Each layer in the encoder and decoder includes several key components: embeddings and positional encoding, layer normalization and residual connections [32], attention mechanism, multi-head attention, and a position-wise feed-forward network. The transformer structure is illustrated in Figure 2 [18], showing the detailed structure of attention and multi-head attention. In the following sections, we give a review of these components.

### 2.1. Embedding Layer and Positional Encoding

The embedding layer maps the features of the input elements, or tokens, into a fixed-dimensional vector with dimension $d_{model}$. This mapping enables the transformer to encode relationships between tokens in the feature space. Since the transformer does not inherently process sequential data like recurrent models, it employs positional encodings to inject information about the order of tokens in the sequence. The positional encoding is generated using the sine and cosine functions of different frequencies according to Equations (1) and (2) [18].

(1) $${PE}_{\left(pos,2i\right)}=sin(pos/{10,000}^{{2i/d}_{model}})$$

(2) $${PE}_{\left(pos,2i+1\right)}=cos(pos/{10,000}^{{2i/d}_{model}})$$

where pos represents the position of the token, i denotes the dimension of the embedding, and $d_{model}$ is the total number of dimensions in the embedding space. This design allows the model to encode information about the relative position of tokens, creating a sense of order in the sequence. The token embeddings and positional encodings are then combined, providing the model with both semantic and positional information about tokens in the input sequence. This approach enables transformers to process long-sequence dependencies, a task which is typically challenging for recurrent frameworks [18]. Moreover, we will later discuss that this unique architecture enables the model to process data in parallel, reducing computation time and making the transformer suitable for large datasets.

### 2.2. Layer Normalization and Residual Connections

During backpropagation, deep neural networks may encounter the vanishing gradient problem, which can result in unstable training. To avoid these issues, transformers benefit from two architectural components: layer normalization and residual connections. Layer normalization normalizes the outputs of each layer, ensuring that the inputs for the next layer have zero mean and a standard deviation of one, which assists in preventing the vanishing gradient problem. Residual connections enable gradients to flow through a branch that bypasses the layer, linking its input directly to its output, thereby ensuring stability. As depicted in Figure 2a, residual connections are applied around the sub-layers before layer normalization [18].

### 2.3. Attention Mechanism

The attention mechanism enables the model to focus on the most relevant parts of the input sequence by assigning attention weights to them. Figure 2b illustrates the self-attention structure. To compute attention for tokens in the input sequence, each token is transformed into three vectors named query (q), key (k), and value (v), all with the same dimension $d_{q}=d_{k}=d_{v}=d_{model}$. For all tokens in the input sequence with length L, these vectors are then packed into Q, K, and V vectors with dimension $L\times d_{model}$. Attention weights are computed according to Equation (3) [18].

(3) $$Attention\left(Q,K,V\right)=softmax\left(\frac{QK^{T}}{\sqrt{d_{k}}}\right)V$$

where term $QK^{T}$ calculates the similarity between Q and K values, and term $\sqrt{d_{k}}$ normalizes the score for gradient stability. The SoftMax function transforms the score into probabilities. Attention is calculated by taking the dot product of these probabilities with the values V, meaning that tokens with higher probabilities receive more attention. Q, K, and V are learned during the training process of the model. Attention mechanisms can take various forms, including self-attention, multi-head attention, and cross-attention [18].

### 2.4. Multi-Head Attention

One of the key factors in the success of the transformer architecture is its ability to perform parallel processing, making it highly suitable for handling large datasets. This is achieved through the multi-head attention mechanism, illustrated in Figure 2c. The feature space of the Q, K, and V matrices are linearly projected and divided into smaller parts. Multi-head attention employs multiple Scaled Dot-Product attention heads, where each head processes a different part of the feature space. The outputs from all attention heads are then concatenated and projected again to reconstruct the fully processed embedding feature space. This mechanism enables the model to process input sequences from diverse attention perspectives in parallel, significantly reducing computational time. Multi-head attention is represented by Equations (4) and (5) [18].

(4) $$MultiHead(Q,K,V)=Concat({head}_{1},\ldots ,{head}_{h})W^{0}$$

(5) $$Where\;{head}_{i}=Attention({QW}_{i}^{Q},{QW}_{i}^{K},{QW}_{i}^{V})$$

where $W^{0}\epsilon R^{d_{model}\times d_{model}}$ is the embedding space, ${QW}_{i}^{Q}$, ${QW}_{i}^{K}$, and ${QW}_{i}^{V}\epsilon R^{d_{model}\times (d_{model}/h)}$ are subdivided embedding spaces for ${head}_{i}$ [18].

### 2.5. Position-Wise Feed-Forward Network

The position-wise feed-forward network is a fully connected feed-forward network (FFN) that is independently applied to each position in the input sequence using a nonlinear activation function (ReLU). It consists of two linear transformations, as shown in Equation (6) [18]:

(6) $$FFN\left(x\right)=max\left(0,xW_{1}+b_{1}\right)W_{2}+b_{2}$$

where $W_{1}$, $W_{2}$, $b_{1}$, and $b_{2}$ are the weight and bias matrices of layers 1 and 2, respectively. The FFNs operate on each position in the sequence individually, meaning the same transformation is applied at each position without any interaction between them. On the other hand, it can be considered as two convolutions with a kernel size of one [18].

## 3. Transformer Models and Their Variants

Since the introduction of the vanilla transformer, transformers have undergone significant evolution. They have retained the attention structure of the vanilla transformer while being modified and expanded across various types and fields. EEG data can be represented as multi-channel time series, images, or graphs, allowing different transformer variants to be explored in EEG analysis studies. The most well-known variants of transformers include the Time Series Transformer, Vision Transformer, and Graph Attention Transformer.

### 3.1. Time Series Transformer

The transformers have demonstrated promising directions in modeling long-range dependencies in sequential data. Time Series Transformers retain the architecture of the vanilla transformer, with certain parameters adjusted for time series analysis, providing valuable insights and achieving successful results in various applications such as classification, anomaly detection, and prediction [33].

EEG is basically a form of time series, inherently stochastic, with both short- and long-term dependency patterns. Indeed, Time Series Transformers benefit from positional encoding to preserve temporal information and the attention mechanism [34], achieving significant results in capturing the long-range dependencies in EEG data. For instance, the literature [35] proposed a transformer model for motor imagery recognition using raw EEG data, reporting high performance. Similarly, a transformer model for single-channel EEG artifact removal was introduced by [34], utilizing the attention mechanism to extract global information from each raw EEG slice and to capture potential artifact patterns.

Furthermore, EEG is a multi-channel time series signal that contains spatial dependencies that correspond to the functional and anatomical structure of brain activity, conventionally depicted through topographic mapping [36]. Preserving these dependencies has been demonstrated to enhance the performance of transformer models [37]. Du et al. presented a temporal–spatial transformer based on a multi-headed attention mechanism for person identification, utilizing raw EEG signals [27]. Another study emphasizing the importance of spatial dependencies in EEG channels was conducted by Tuncer et al., who introduced a neonatal seizure detection model that focuses on identifying the importance of different EEG channels rather than directly analyzing the signal amplitudes [38]. A common strategy to effectively capture the spatial dependencies of EEG signals is to preserve the spatial configuration of EEG channel locations [39,40]. 

In the following section, we categorize transformer-based EEG analysis studies into two distinct model approaches: end-to-end models, which process raw EEG data directly without feature extraction, and feature-driven models, which use extracted features.

#### 3.1.1. End-to-End Model

**Figure 3:**
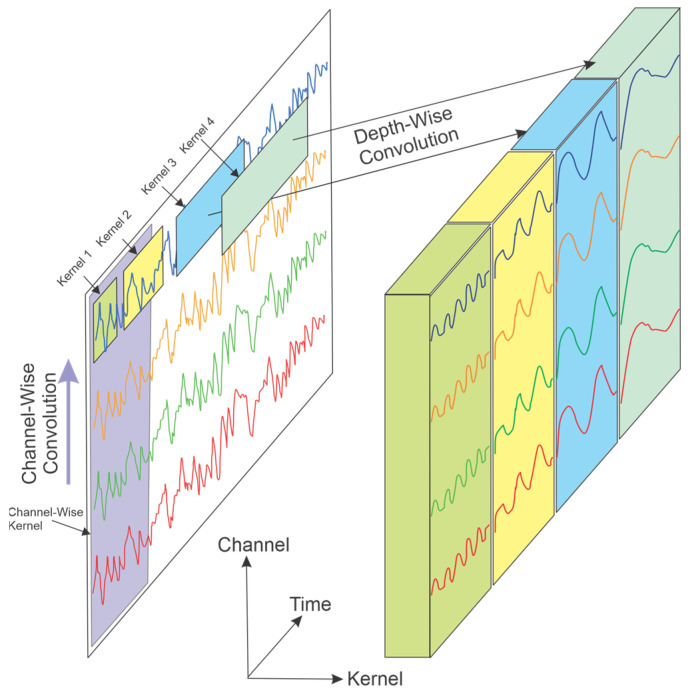
The structure of convolutional layers in end-to-end models [45].

End-to-end models benefit from convolutional layers, where the transformer directly learns the temporal and spatial dependencies of EEG data and automates feature extraction. These models utilize temporal or depth-wise and spatial or channel-wise convolutional layers, depending on the study’s objective and the focus on the temporal or spatial dependencies of the EEG patterns [41]. The model structure and layers are defined based on the experimental design. Depth-wise convolutional layers are employed to capture temporal–spectral dependencies [42], while spatial convolutional layers are effective in capturing channel dependencies or brain region-based dependencies. The structure of the convolutional layers is depicted in Figure 3. Luo et al. applied a temporal convolution layer, an encoder, and a classifier to develop an end-to-end model for motor imagery [43]. In a similar approach, a convolutional transformer model that integrates both local and global features for EEG classification was proposed by [44]. Their model incorporated a convolution module to capture low-level local features using one-dimensional temporal and spatial convolution layers, a self-attention module to extract global dependencies, and a classifier composed of fully connected layers.

Depth-wise convolution: The depth-wise convolution layers incorporate different temporal kernel sizes, enabling the transformer to learn the various spectral–temporal representations of EEG signals [46]. As illustrated in Figure 3, these kernels are designed to effectively extract temporal patterns from the signal, capturing both short- and long-term dependencies. The length of the temporal kernel corresponds to the EEG frequency ranges, meaning that different kernel sizes extract different EEG frequencies. Larger kernels extract low-frequency features, whereas smaller kernels extract high-frequency features. Consequently, multi-scale temporal kernels provide a wide range of frequency representations, allowing the model to learn more context-related information, which can lead to improved model performance [45]. In a study, the literature [42] proposed an emotion recognition model incorporating depth-wise convolutional transformer encoders, achieving accuracies of 93.83% and 83.03% for subject-dependent and subject-independent experiments, respectively. Furthermore, a driver fatigue recognition study utilized a multi-scale convolutional transformer, reporting strong performance [47]. In another related study, ref. [40] applied a multi-scale convolutional transformer for decoding mental imagery across the spatial, spectral, and temporal domains.

Channel-wise convolution: The channel-wise convolutional layer consists of multi-scale one-dimensional spatial kernels that allow the transformer to learn the spatial dependencies of EEG signals, with kernel sizes corresponding to the spatial locations of the EEG channels. The global kernel has a size equal to the total number of channels, making it capable of learning global spatial information across all channels [48]. Using this approach, Peng et al. employed a transformer model for emotion recognition, proposing a channel attention mechanism to capture the contributions of individual EEG channels [49]. Similarly, Yauri et al. investigated a transformer model for epileptic seizure detection, consisting of convolutional layers for channel fusion [50]. In another study, a transformer-based SSVEP-BCI model was proposed by Wan et al., consisting of a one-dimensional convolutional layer for automatically extracting channel-wise features [41]. Furthermore, a transformer model for depression identification was suggested by Hou et al., incorporating a channel modulator that dynamically adjusts the contribution of each electrode channel [51].

Relationships between specific regions of the brain are captured by the region-wise kernel, which shares convolutional kernels across the channels within a given region [2]. For spatial kernels to be applied effectively, the sequence of channels in the input EEG samples must be arranged according to their channel or region locations. This arrangement ensures that the kernel weights are shared among adjacent channels or regions, allowing for the effective learning of spatial dependencies within the EEG data.

**Figure 4:**
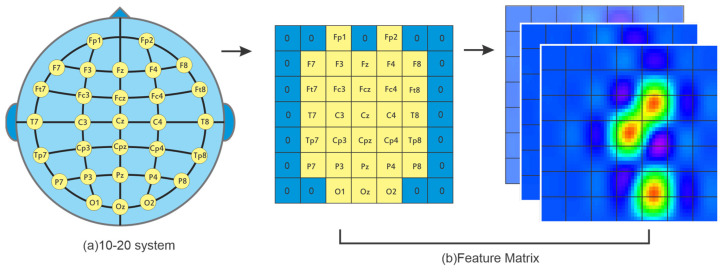
Preserving spatial EEG information based on the 10–20 system to capture regional dependencies.

For instance, Du et al. employed a transformer model for emotion recognition, where the extracted EEG features were spatially mapped into a matrix based on the electrode locations in the international 10–20 system of electrode placement [52]. A similar study was proposed by Gong et al., which preserved the spatial relationship of EEG electrodes by projecting EEG features [39]. In a region-based approach, Lee et al. introduced a transformer architecture with two parallel branches, each processing the frontal and temporal lobes individually [53]. Similarly, a transformer model for motor imagery classification was suggested in [54], comprising local and global convolutional layers that individually process three brain regions: the left hemisphere, right hemisphere, and motor region. Figure 4 illustrates the feature mapping based on electrode locations, enabling the application of region-wise kernels.

#### 3.1.2. Feature-Driven Model

Feature-driven models rely on traditional feature extraction techniques. In this approach, transformer models receive the extracted features as an input sequence [55]. The feature extraction process involves preprocessing steps for raw EEG data, including band-pass filtering, artifact removal, Z-score normalization [27], segmentation, and feature extraction. The selection of EEG features in transformer models varies depending on the study’s objectives. For example, Oh Shu Lih et al. developed an epilepsy detection transformer model by investigating the Pearson Correlation Coefficients (PCCs) extracted from 5-s epochs as the input sequence to the model [56]. Zeynali et al. employed Power Spectral Density (PSD) features to capture both the temporal and spectral dependencies of EEG in a BCI transformer network [57]. An attention network was created by Xinyue Zhong et al. to model the asymmetric property of the brain’s emotional response using Differential Entropy (DE) [58]. In the same vein, Yang Dai et al. introduced a sleep classification transformer model that utilizes the short-time Fourier transform (STFT), treated as the input vector T×F, where T and F represent the time and frequency dimensions, respectively [59]. Similarly, Yan et al. developed a seizure prediction model consisting of three transformer branches, each processing STFT data in a channel-wise, frequency-wise, and step-wise manner, followed by a gating layer to fuse the results [60].

### 3.2. Vision Transformer

**Figure 5:**
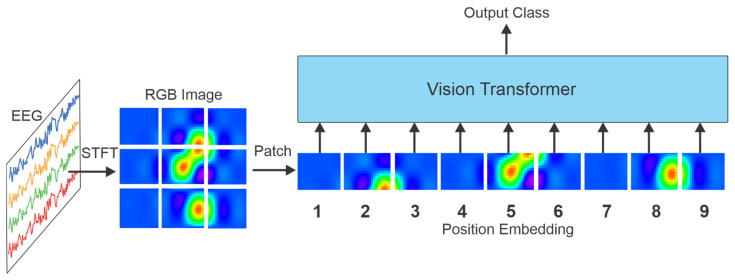
In a ViT architecture, the input RGB image is broken down into small patches, and position embeddings are added to preserve the spatial information of the patches in the entire input image.

Dosovitskiy et al. introduced the Vision Transformer (ViT) in 2020 by adapting the vanilla transformer architecture for image analysis [22]. The Vision Transformer structure is shown in Figure 5. The ViT divides an image into fixed-size patches serving as input tokens. These patches are linearly embedded, and positional encodings are applied to preserve the spatial information within the image.

In detail, based on [22], the vanilla transformer takes one-dimensional sequences of token embeddings as input. To adapt two-dimensional images, an image X with dimensions H×W×C, where (H×W) represents the resolution, and C is the number of channels, is split into sequence square patches of size P×P. Each patch is flattened into a vector, creating a total number of $N=\frac{HW}{P^{2}}$ patches, which is the effective input sequence length of the ViT. Since the transformer uses a fixed vector size across all its layers, the flattened patches are mapped to one-dimensional vectors through a learnable linear projection. A set of standard learnable one-dimensional positional embeddings is then added to the patch embeddings to retain positional information. The transformer encoder consists of multi-headed self-attention, normalization, and Multilayer Perceptron (MLP) layers. The MLP layers operate locally, while the self-attention layers are global, capturing dependencies across the entire image, enabling ViT to effectively model complex visual patterns. Although ViTs require large-scale datasets to outperform traditional vision models, their multi-head architectures make them more computationally efficient and a successful alternative to models such as CNNs [22].

**Figure 6:**
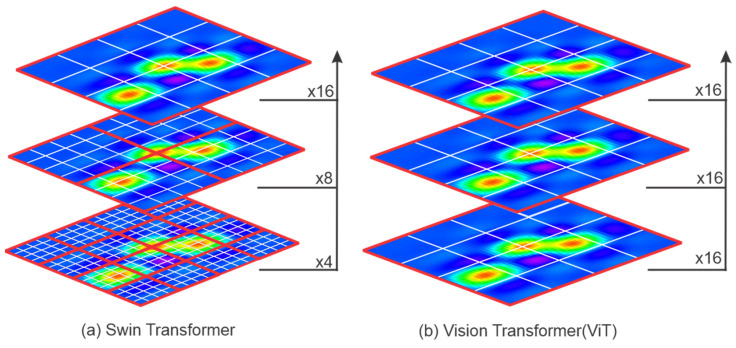
(**a**) Swin Transformer; (**b**) Vision Transformer (ViT) [25].

The large variation in image content and the high resolution of pixels are two main challenges in adapting the vanilla transformer from text to images. To address these challenges, Ze Liu et al. introduced the Swin Transformer in 2021 [25]. The Swin structure is designed to hierarchically reduce the number of tokens by a downsampling factor of two per stage. It uses a local attention mechanism within non-overlapping shifted windows to capture both local and global dependencies. This window-based attention significantly reduces computational complexity, making it more efficient for high-resolution image analysis. Similarly to the ViT, the Swin Transformer processes an input RGB image (H×W×(C=3)) by first splitting it into non-overlapping patches. Figure 6 illustrates the different approaches for the Swin Transformer and the ViT.

**Figure 7:**
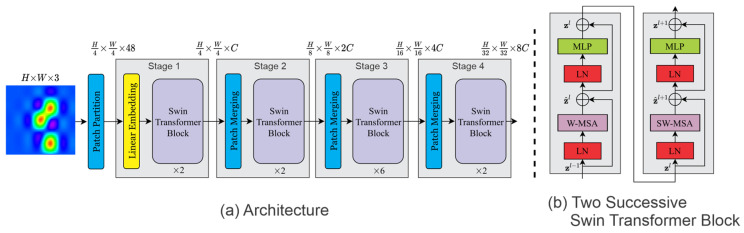
(**a**) Swin Transformer architecture; (**b**) Two successive Swin Transformer blocks [25].

Each patch is considered a token and forms a feature vector by concatenating the raw RGB pixel values. For a patch size of 4×4, the feature dimension is 4×4×3=48, and the total feature dimension is $\frac{H}{4}\times \frac{W}{4}\times 48$. Figure 7 provides a simplified overview of the Swin Transformer architecture. The Swin architecture (Figure 7a) involves four stages:

- Stage 1: A linear embedding layer projects the raw features into a C-dimensional space. Two transformer blocks with modified self-attention (Swin Transformer blocks) process the patches. The transformer blocks maintain the number of tokens to $\left(\frac{H}{4}\times \frac{W}{4}\right)$.
- Stage 2: In a hierarchical structure, as the network goes deeper, the number of tokens is reduced by patch merging layers. The first patch merging layer concatenates features from 2×2 neighboring patches into 4C-dimensional features. This reduces the number of tokens to $\frac{H}{8}\times \frac{W}{8}$ with a downsampling factor of 4. At output, the linear embedding layer dimension is set to 2C. The Swin Transformer blocks further transform the features at a lower resolution, completing “Stage 2”.
- Stage 3 and stage 4: This process repeats progressively.

The Swin Transformer benefits from a hierarchical structure, where feature vectors are progressively downsampled in deeper layers, similar to CNNs, allowing it to capture both fine-grained and abstract features. This architecture allows the Swin Transformer to outperform both the vanilla ViT and CNNs on large-scale datasets [25].

#### EEG Vision Models

There are several approaches to transforming EEG data into images, which focus on different aspects of the data, including temporal, spatial, and spectral features. Figure 5 represents a typical ViT architecture that processes STFT images extracted from EEG data. Each approach requires a specific transformer architecture design. For instance, Chen et al. proposed a ViT model for Alzheimer’s disease prediction by utilizing a raw EEG signal plot as an input image [61]. In another study [62], they introduced a ViT model for decoding the user’s movement preparation using Continuous Wavelet Transform (CWT) images extracted from EEG signals. In many studies employing the ViT, spectral transformations are typically applied to convert EEG signals into image formats, and multi-channel ViTs are used to process images from individual EEG channels. For example, Dong et al. proposed a multi-channel ViT for seizure prediction, utilizing the Stockwell transform for the time–frequency representation of multi-channel EEG [63]. In [64], A Bi-branch ViTs model for emotion recognition was developed, where each branch processed spatio-temporal and spatial-spectral images while preserving electrode locations. Moreover, Hussein et al. employed a multi-channel ViT for epileptic seizure prediction, utilizing scalogram images extracted from segmented multi-channel EEG [28].

There are some studies utilizing the Swin Transformer to extend the applicability of the ViT. A Swin Transformer model for drowsiness recognition. For example, in a recent study by Zhang et al., data augmentation (DA) and self-supervised learning techniques were integrated to enhance recognition accuracy and generalization [65]. In another study, ref. [66] developed a multi-scale Swin Transformer model that integrates parallel convolution and attention mechanisms for enhanced EEG-based cognitive load assessment, employing short-time Fourier transform (STFT) to construct multi-dimensional EEG feature representations. To capture the temporal, spectral, and spatial features embedded in EEG data for motor pattern classification, Han Wang et al. developed a model that combines a channel-attention mechanism with Swin Transformer, utilizing Common Spatial Patterns (CSPs) as the feature extraction method [67]. Likewise, in an emotion recognition study, Cai et al. employed a Swin Transformer with EEG images derived from DE [68].

### 3.3. Graph Attention Transformer

The Graph Attention Transformer (GAT), introduced by Veličković et al. in 2017, is a neural network model that applies attention mechanisms to graph-structured data, enabling more flexible and adaptive learning of node relationships [23]. By leveraging a self-attention mechanism, GAT computes attention coefficients for each edge in a node’s neighborhood, dynamically determining the importance of each neighbor during the learning phase. This allows the network to focus on the most relevant connections, enhancing its ability to learn complex patterns in the graph. Unlike traditional graph convolutional networks (GCNs), which use fixed graph adjacency matrices, GAT learns edge weights directly, offering more robustness. Multi-head attention further enriches the model and makes GAT effective for tasks like node classification and link prediction.

To better understand the GAT architecture, it is useful to begin with the concept of CNNs and their extension to GCNs. In a CNN, convolution kernel weights are applied to the input data, effectively modeling relationships between local regions. Similarly, in graph neural networks, edge weights can be interpreted as convolution kernel weights, representing the influence of one node on another. The adjacency matrix A is used to indicate the connectivity between nodes, where an entry of 1 indicates an edge, and 0 indicates no edge. In GCN application, A is commonly a fixed matrix and pre-defined. GAT introduces a learnable attention mechanism that dynamically assigns edge-specific weights, replacing the fixed A term with learned attention coefficients. This mechanism enables GAT to prioritize important edges, leading to more expressive node representations. The resulting flexibility improves performance in complex and heterogeneous graphs.

**Figure 8:**
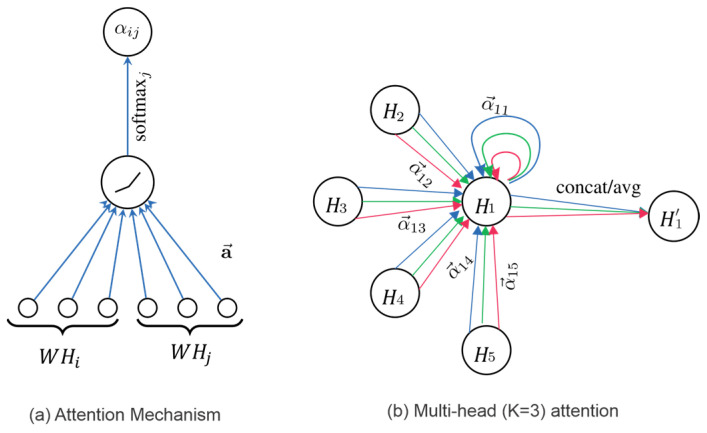
(**a**) Graph Attention Transformer (GAT); (**b**) multi-head attention [23].

Nodes in the GCN are updated as follows: $H^{\left(l+1\right)}=\delta (D^{-\frac{1}{2}}A^{\sim }D^{-\frac{1}{2}}H^{l}W)$, where $A^{\sim }=A+I$ is the adjacency matrix A added with identity matrix I to include a self-loop for nodes, D is defined as $D_{ii}=\sum_{j}A_{ij}^{\sim }$ to normalize $A^{\sim }$, δ is the sigmoid activation function, the features matrix is at layer I, and W is the weight matrix of the layer. The GAT extends the GCN by introducing a learnable attention mechanism that assigns different importance to neighbors during feature aggregation. The updated formula for GAT is expressed as follows: $H_{i}^{\left(l+1\right)}=\delta (\sum_{j\epsilon N\left(i\right)\cup \left\{i\right\}}\alpha_{ij}^{\left(l\right)}W^{\left(l\right)}H_{j}^{\left(l\right)}),$ where $H_{i}^{\left(l+1\right)}$ is the updated feature vector for node i at layer l+1, W is the weight matrix, $\alpha_{ij}^{\left(l\right)}$ is the attention coefficient, and δ is the sigmoid activation function. The attention mechanism affects the aggregation step by replacing the static normalization factor $D^{-\frac{1}{2}}A^{\sim }D^{-\frac{1}{2}}$ in GCN with dynamically computed attention coefficients $\alpha_{ij}^{\left(l\right)}$. The learned attention coefficients $\alpha_{ij}^{\left(l\right)}$ dynamically adjust the influence of neighbors, making GAT more expressive than GCN. By applying multi-head attention, GAT further captures diverse relationships between nodes. GAT computes multiple independent sets of attention coefficients $\alpha_{ij}$ through multiple attention heads. Each head processes the graph separately and generates its own node features as shown in Figure 8 [23].

#### EEG Graph Attention Models

EEG signals are recorded by placing electrodes on the scalp, forming a correlated, fixed, and regular arrangement of electrodes, which makes EEG data ideal for graph representation [69]. In this undirected graph, each electrode is presented as a node, and the relation between electrodes is represented as edges, with the adjacency matrix A capturing these relationships. The matrix A is learnable and can be updated during training. Common methods for calculating the adjacency matrix A include correlation-based methods, typically using PCC [70], coherence-based methods, which measure the correlation between two signals in the frequency domain, often across different frequency bands, Granger causality, which assesses whether the past values of one signal can predict the future values of another, and phase-locking value, which measures the phase synchronization between EEG signals [71].

Li et al. proposed an emotion recognition GAT model that integrates both spatial and temporal attention mechanisms to capture dynamic connections between brain regions. The authors reported that the adjacency matrix learned by the model provides a more accurate graph representation, as it is adaptively updated through spatial attention during the training process [72]. Similarly, Jia et al. developed a GAT model for fatigue driving detection, which dynamically processes the extracted adjacency matrix as a graph representation of EEG data [73]. In another study, a transformer-based seizure prediction model was introduced by Yifan Wang et al., employing a point-wise dynamic multi-graph convolution network to dynamically learn deep graph structures from the extracted features [74]. In another study, Lian et al. suggested an epileptic EEG classification model consisting of a graph neural network to uncover the underlying relationships between multi-channel EEG signals, followed by a transformer layer to capture the dependencies across the channels [29].

### 3.4. Hybrid Models

**Figure 9:**
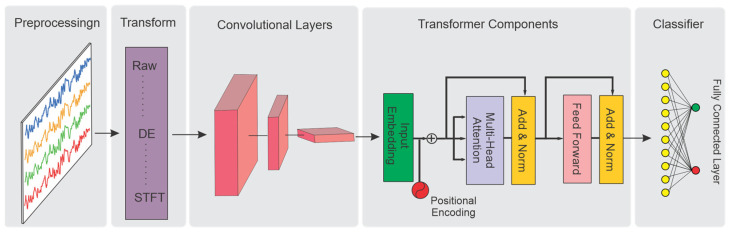
Hybrid transformer, including components and layers.

Hybrid transformer models are end-to-end architectures that integrate traditional machine learning techniques for local feature extraction with transformers to capture global dependencies. Figure 9 provides an overview of a hybrid transformer architecture, comprising various layers and components, including a preprocessing stage, transformation layers, convolutional layers, transformers, and a fully connected classifier. These models leverage the strengths of traditional methods, such as CNNs for extracting localized features, and transformers are utilized to capture long-range dependencies. This combination makes hybrid transformer architectures particularly effective for EEG signal analysis, where spatial, temporal, and spectral features exhibit both short- and long-term dynamics. Depending on the specific objectives of a study, the architecture of hybrid models may vary. In general, hybrid models typically integrate convolutional layers with attention mechanisms, followed by classifier layers.

For instance, Sun et al. proposed an end-to-end hybrid architecture for seizure detection that combines a CNN for feature extraction, the encoder component of a transformer for capturing dependencies, and an MLP head as the classifier [75]. Similarly, a hybrid model for seizure prediction was introduced by [76], integrating CNNs and transformers. The model utilized STFT as input features, aiming to capture local features through CNNs and long-range dependencies using transformers. Furthermore, Sun et al. designed a hybrid model aimed at consciousness detection by utilizing a pool of spectral, complexity, and connectivity features [77]. This innovative combination proved effective in distinguishing different states of consciousness. In another study, a hybrid model for seizure detection was proposed by Tian et al., in which their model included CNNs and a transformer encoder. Brain connectivity, represented as image-like features, was fed into a series of CNN layers and transformer encoders [70]. Likewise, Wei Zhao et al. employed a hybrid motor imagery classification model. Their architecture consisted of CNN layers dedicated to extracting local and spatial features, a transformer encoder layer for capturing the global dependencies of EEG high-level features, and fully connected layers for final classification. This model achieved high classification accuracy, showcasing the potential of hybrid architectures in motor imagery tasks [16]. In another notable development, in [78], an advanced hybrid neural network integrating CNN layers with a transformer decoder was introduced, achieving a significant improvement in cross-subject EEG motor imagery classification accuracy.

There are several studies aimed at improving model performance by combining the effectiveness of transformer encoder layers with other traditional structures. Zhao et al. proposed a hybrid seizure detection model, utilizing a CNN to extract local features, a transformer to capture global features, and a feature coupling block to fuse the information interactively [79]. This approach further enhanced the model’s ability to detect seizures with high precision. Moreover, Yao et al. introduced a hybrid emotion classification model, consisting of two parallel transformer encoders. These encoders receive raw EEG data in temporal and spatial arrangements to capture temporal and spatial features. The concatenated features are then fed into a CNN layer for classification, resulting in improved emotion recognition performance [80]. Similarly, Si et al. investigated an ensemble model for emotion recognition, combining a pure CNN model with a cascaded CNN–transformer hybrid model, thereby improving the recognition accuracy [81].

## 4. Applications of Transformer Models in EEG Analysis

**Figure 10:**
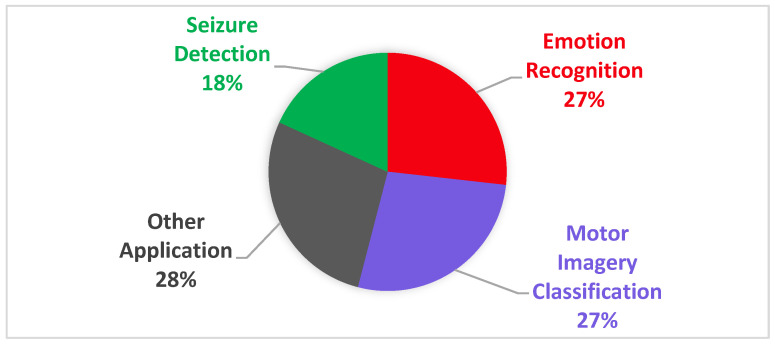
Comparison of applications in transformer-based EEG analysis used in the studies discussed in this review.

Among the studies reviewed in this paper, the most frequent applications are categorized into three domains: motor imagery (MI) classification, emotion recognition, and seizure detection. On the other hand, less common studies are grouped under “other applications”, including tasks such as mental workload estimation, sleep stage classification, person identification, and the diagnosis of conditions such as depression and dementia. Figure 10 presents the usage percentage of transformer applications in EEG analysis, as employed in many of the research papers in this review. Table 1 provides an overview of the dataset abbreviations and their corresponding full names used in the papers reviewed in this survey. The following section briefly reviews recent transformer applications. 

### 4.1. Motor Imagery (MI)

Recent studies demonstrate a rapid increase in the application of transformers in EEG-based BCI. Motor imagery, which refers to the mental simulation of movement without actual execution or muscle activation, is the most common BCI paradigm [82]. Indeed, MI-based BCI facilitates the direct control of computer applications via brain activity, with primary applications in rehabilitation and assistive technologies [83]. The reported performance for transformer models in motor imagery (MI) recognition is state-of-the-art compared to previous deep learning models. This is demonstrated in the literature [84], where a transformer-based model was used to classify EEG signals in MI tasks for spinal cord injury patients. A hybrid transformer–GCN model, as suggested in [71], improved MI-EEG signal classification, achieving high accuracy with just 2 s of data and demonstrating significant promise for real-time applications.

Furthermore, transformers enable end-to-end architectures to capture complex features and enhance performance, with several studies demonstrating the benefits of this approach [85]. A study employed a hybrid end-to-end transformer model based on the ViT, achieving superior MI decoding performance [46]. The impressive performance of transformer-based models suggests that MI studies are advancing to the next level, aiming to improve cross-subject performance. A study reported classification accuracies of 81.33% and 86.23% in cross-subject experiments utilizing the BCI Competition IV 2a and 2b datasets [86].

Moreover, several studies focus on enhancing the accuracy and generalization of transformer models in this domain. For instance, Hameed et al. utilized a transformer architecture model with a self-attention mechanism to improve classification generalization. Their findings highlighted greater stability in both subject-dependent and subject-independent settings [87]. Similarly, a transformer model integrating data augmentation was proposed by Chen et al. to improve classification accuracy by capturing both local and global features. They reported an average accuracy increase of 7.29% with data augmentation compared to without it [48]. Another study on the same dataset applied data augmentation and reported an approximately 3.0% improvement over similar works [88].

Based on the reviewed papers, most transformer-based BCI research focuses on MI and, to a lesser extent, on other EEG classifications, such as SSVEP-based BCIs. Furthermore, as partially indicated in Table 2, hybrid models and Time Series Transformers emerge as the most successful architectures. Performance results show that hybrid models exhibit superior classification performance. SSVEP refers to a steady-state evoked potential in response to visual stimuli flickering at specific frequencies. In this field, Ding et al. employed a transfer model as an asynchronous classification system for an SSVEP-based BCI, enhancing robustness and flexibility in human–machine interaction systems [89]. Similarly, a transformer algorithm was suggested by Qin et al., designed to holistically capture the spatio-temporal information of SSVEP [90].

### 4.2. Emotion

Given the dynamic nature of emotion, classification models must be designed to capture the intricate spatio-temporal dependencies inherent in EEG signals [15]. Transformers offer a framework for effectively capturing the inherent temporal and spatial characteristics of EEG signals. The complex patterns of EEG in emotional states require considering all spatial, temporal, and spectral features. Some studies employ transformer models that integrate these features to improve classification performance for emotion recognition tasks.

Furthermore, the dynamic nature of emotion patterns can be effectively captured by the attention mechanism, and spectral information can also be incorporated through spectral transformations such as wavelet transformation [91]. A study in this context utilized a graph-based structure to enrich spatial information and incorporated it with temporal information [69]. Likewise, Guo et al. proposed a model that used a graph convolutional network to obtain channel-wise enhanced features and a cross-transformer to capture long-range dependencies across multiple temporal scales [92]. Graph Attention models efficiently capture spatial connectivity and learn spatial features more effectively, thereby enhancing feature representation [93]. With the success of attention mechanisms, recent studies have demonstrated significant improvements in EEG emotion classification performance by identifying the most relevant EEG channels. Notably, in [94], a spatial channel attention mechanism was employed on the DEAP dataset. Moreover, the feature set selected by the attention mechanism can effectively enhance the performance of the transformer model in classifying emotional EEG sequences [95].

Thanks to hybrid transformer architectures utilizing convolutional layers, which replace traditional feature extraction procedures, end-to-end system models have become an interesting topic among recent transformer-based studies [96]. Zheng et al. proposed an end-to-end model that directly processes raw EEG signals by a data-driven approach [17]. However, hybrid models indicated strong performance in a feature-driven manner [52]. In particular, the hybrid model proposed in [97] utilized a depth-wise convolution layer, achieving robust performance in cross-subject emotion recognition. In addition, a proposed transformer-based method in [98] employed attention mechanisms to amplify emotion-related features and minimize emotion-unrelated features in the brain. Recent research in emotion recognition has utilized various forms of transformer models. As indicated in Table 2, hybrid architectures are of greater interest. Furthermore, hybrid and vision-based models have demonstrated superior classification performance.

Given the promising results of transformers in emotion recognition, recent studies have focused on leveraging transfer learning to boost performance. For instance, one study proposed a dual transfer learning method and reported a remarkable 98.69% accuracy on the SEED dataset [99]. Similarly, another recent study implemented a domain adaptation method to improve transfer learning capabilities. They reported accuracy improvements in intra-subject experiments of 54.70% and 43.70% on the SEED and SEED-IV datasets, respectively [100].

### 4.3. Seizure

Seizure is identified through abnormal EEG patterns, and the accurate detection of seizures is essential for effective treatment plans and monitoring epilepsy progress. EEG-based seizure detection is particularly challenging due to patient-specific factors, variability in seizure types, and noise [101]. Recent advancements in deep learning models, particularly those utilizing Vision Transformers, have significantly enhanced seizure detection model performance [63]. For instance, Yuan et al. proposed a CNN layer combined with the ViT and an attention mechanism to predict epileptic seizures from EEG data, achieving improved accuracy by leveraging local and global feature extraction capabilities [102]. Similarly, studies utilizing a ViT model have demonstrated a sensitivity of 94.70% [103].

The complexity of seizure patterns and irregular temporal fluctuations requires models capable of identifying hidden spatial and spectral patterns. Hybrid models effectively address this challenge by encompassing a diverse feature space within an end-to-end architecture. A study employing a transform-based depth-wise convolutional model reported a sensitivity of 94.27% on an unseen dataset [104]. Likewise, a study using a hybrid model applied STFT to extract three-dimensional EEG features, encompassing time, channel, and frequency dimensions, and reported an average sensitivity of 98.24% on the CHB-MIT dataset [105]. The study [106] introduced a novel framework for EEG-based patient-specific seizure prediction called the Spatial–Temporal Hypergraph Attention Transformer. The goal was to enhance seizure prediction accuracy by leveraging both temporal and spatial dependencies within EEG signals, resulting in a sensitivity of 94.18% on the CHB-MIT dataset. According to the literature, hybrid and Vision Transformer models are commonly employed in seizure detection studies. Recent studies, as indicated in Table 2, demonstrate that hybrid models exhibit superior performance compared to other models.

GAT has demonstrated strong capabilities in capturing spatio-spectral information, leading to high-performance seizure detection [107,108]. As reviewed, transformers have significantly outperformed traditional deep learning models, and recent studies have focused on extending the application of transformers to the transfer learning approach, with the goal of improving cross-patient performance [109], model generalization, and achieving robust performance with small datasets. In a study, a modified ViT model investigated for cross-subject seizure classification, capable of handling small labeled datasets or other investigations, utilized mutual distillation between raw EEG data and its wavelet representations to enable effective knowledge transfer [110], highlighting transfer learning as a future direction for EEG-based transformer studies [111].

### 4.4. Other Applications

Transformer models have advanced EEG analysis by effectively capturing complex signal patterns and enhancing model performance. Standardized databases have furthered research in key areas such as BCI, emotion classification, and seizure detection. Recently, the application of transformers has expanded to broader EEG-based tasks, including sleep staging, mental workload assessment, and diagnosing neurological conditions like Alzheimer’s disease, depression, and dementia. The following section briefly reviews these applications.

EEG signals, due to their high variability and noisy nature, require advanced reconstruction and denoising methods. Transformer models have demonstrated strong potential in handling missing EEG segments, outperforming other deep learning methods. The successful application of transformers in denoising provides an exciting field where the attention mechanism helps capture dependencies while ignoring less important parts, offering a new approach to EEG denoising. Transformers have demonstrated effectiveness in EEG denoising by learning to map noisy input signals to their noise-free signals. For example, a denoising transformer architecture was introduced by Pu et al., where an EEG noise-added signal is considered as the input, and noise-free EEG is used as the output. They reported 18% and 11% improvements in correlation coefficients for the removal of EOG and EMG artifacts, respectively [112].

Furthermore, CNN architectures provide local and global feature extraction, which can improve denoising when combined with the attention mechanism. Jin Yin et al. introduced a GAN-guided parallel CNN and transformer network for EEG denoising. The CNN and transformer blocks capture local and global temporal dependencies, and a discriminator is used to identify and correct mismatches between clean and denoised EEG signals [113]. Another study employed a transformer model to address missing EEG amplitude data, outperforming traditional methods such as Zero, Mean and KNN imputation [114].

Sleep stage classification is a key factor for sleep analysis, and several studies have demonstrated strong performance using transformers for sleep tasks. One such study utilizing a hybrid transformer reported accuracies of 89.2%, 86.6%, and 89.7% on the SleepEDF-20, SleepEDF-78, and SHHS datasets, respectively [115]. Likewise, another study reported accuracies of 86.0%, 82.7%, and 85.1% on the same datasets, respectively, utilizing a hybrid transformer model [116].

Recent research has demonstrated the potential of transformer models in improving the accuracy of mental disease diagnosis [117]. A study integrating ViT and STFT achieved an Alzheimer’s disease classification accuracy of 92.59 ± 2.3% [118]. In addition, a transformer model study for depression identification reported classification accuracies of 94.42% and 94.96% on the study dataset and MPHC2, respectively [51].

Fatigue detection plays a critical role in ensuring driver safety, and recent research has investigated the application of transformer models for decoding fatigue from EEG signals. One study, with a cross-subject EEG aim, reported accuracies of 91.19% and 79.68% for the SEED-VIG and SAD datasets, respectively [119]. Similarly, an end-to-end transformer model reported classification accuracies of 66.03% and 83.52% on the same datasets [47]. In a related study, Lu et al. proposed a ViT for jump motion intention recognition [120].

## 5. Challenges and Future Research Directions

Despite their success in domains such as natural language processing and image classification, transformers encounter significant challenges and limitations when applied to EEG analysis. First, the foremost limitations are data scarcity and generalization issues. Second, transformers require large datasets to perform effectively. On the other hand, EEG data collection is often time-consuming and costly, resulting in limited size for most available EEG datasets. Furthermore, even under controlled experimental conditions, EEG signals are noisy and highly variable, influenced by factors such as individual differences in brain structure, anatomy, personality traits, and past experiences. This high variability in the EEG signal causes poor model generalization and limits their practical application. Several studies aim to overcome these limitations through two main approaches: data augmentation and transfer learning. Moreover, due to their inherent complexity, transformer models can be challenging to interpret in terms of data flow and architectural design. To enhance model transparency, ablation studies are conducted to assess the impact of individual components.

### 5.1. Data Augmentation (DA)

Data augmentation (DA) is a technique that is widely used to address the challenge of overfitting in deep learning models trained on small datasets [89]. Indeed, when the training set is limited in size and derived from a small subset of the original data, the model is prone to overfitting, thereby compromising its generalization capability. DA increases the size and diversity of the training set by applying various transformations. A primary EEG DA technique is segmentation [121]. EEG segmentation involves partitioning a non-stationary signal into shorter, quasi-stationary segments that maintain consistent statistical properties [122]. This process is particularly useful for augmenting the number of labeled data samples.

**Figure 11:**
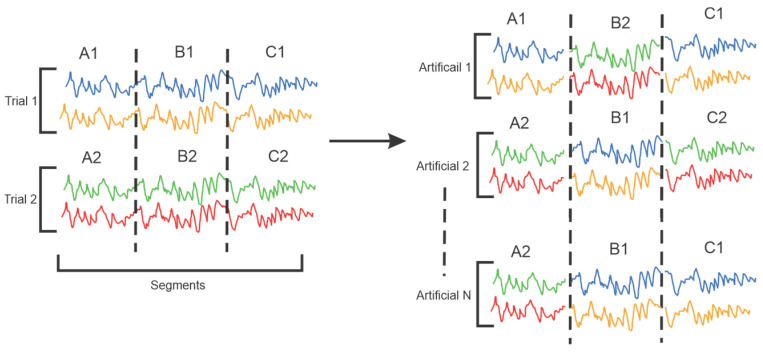
Segmentation and Recombination method as DA technique [16].

Mulkey et al. proposed a ViT model for delirium prediction. They transformed the raw overlapping windows of EEG into images as input and utilized overlapping windows as a data augmentation technique [123]. Similarly, Zhao et al. proposed a method called “Segmentation and Recombination” (S&R) to augment training datasets. The principle of this method is illustrated in Figure 11. This method involves dividing each EEG training trial into several non-overlapping segments. New artificial trials are then created by randomly selecting segments from different training trials of the same category while maintaining temporal sequence, thereby increasing the diversity of the training data without losing the temporal structure of the original signals [16].

**Figure 12:**
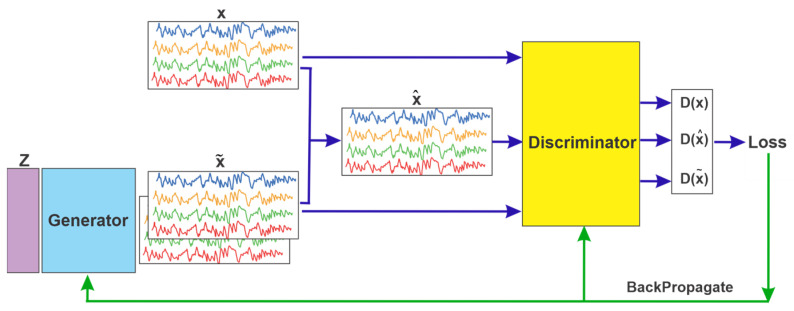
Generative adversarial network structure [125].

Generative adversarial networks (GANs) are another DA method used to generate synthetic data [124]. For example, Yudie Hu et al. applied a GAN to generate new data by modeling the distribution of the original data. The model consists of a generator and a discriminator. The generator samples from a low-dimensional distribution and maps these samples to a high-dimensional space where real data exist, while the discriminator evaluates the output produced by the generator. Through iterative training, both components improve, enhancing the generator’s ability to produce realistic data and the discriminator’s ability to distinguish between real and generated data. This process is depicted in Figure 12, where z is sampled from Gaussian noise, x represents the original data, $\tilde{x}$ denotes a stochastic mixture of real and generated data, and $\tilde{x}$ represents the generated data. During training, a noticeable distribution gap exists between the generated and original data, requiring several optimization iterations for the GAN to converge. This study reported enhancements in recognition accuracies by 2.49%, 2.59%, and 1.14% across three deep learning models [125].

An increasing number of EEG analysis studies focus on enhancing transformer generalization through DA techniques. For instance, Luo et al. implemented a mirroring effect by reversing the channel order in EEG data and claimed that, as an augmentation technique, this effect facilitates a user-independent model [43]. Moreover, ref. [57] introduced an ensemble transformer framework, suggesting that these strategies aggregate transformer outputs to enhance generalization performance. A novel data augmentation approach called EEG mask encoding was proposed by Ding et al. to mitigate model overfitting. They reported significant enhancements in the average classification accuracy of the transformer model, achieving improvements of 3.18% and 11.09% on two datasets [89]. Another study [126] introduced an EEG signal generation model that leverages frequency–spatial correlation. The method begins with forward diffusion, adding noise to real EEG samples. Subsequently, the proposed model is employed to denoise and reconstruct the original EEG signals. The authors reported performance improvements of 0.96% to 1.91% compared to other existing methods. Although DA approaches show performance improvement in some cases, their impact is occasionally limited, highlighting the need for other methods such as transfer learning.

### 5.2. Transfer Learning

Recently, transfer learning has gained increasing interest in EEG-based deep models as a solution to address the challenge of limited labeled data by leveraging knowledge from related domains or tasks. It enables models to be pre-trained on large, available EEG datasets or datasets from other domains with similar characteristics. This approach offers significant potential in improving the performance and generalization of transformer models. For instance, Hu et al. investigated the applicability of transfer learning techniques and model inputs for different deep learning structures in an epilepsy prediction study. The authors reported that their method significantly improves model performance and is more suitable for transformer models compared to traditional CNN-based models [127]. Some studies aim to benefit from the successful results of pre-trained deep models and apply them to EEG studies. For example, Nogales et al. proposed integrating a pre-trained transformer with EEG for Parkinson’s disease diagnosis. They adapted BERT models, considering the similarity between text and EEG signals [128]. In another study, an approach to language model (LM) architectures for the development of EEG modeling was suggested by Kostas et al., adapting an automatic speech recognition approach to learn the compressed representations of raw EEG signals. Their results demonstrated that a single pre-trained model is capable of modeling raw EEG sequences, even when recorded with differing hardware, across different subjects, and for multiple tasks [129].

Domain transfer involves transferring knowledge from a source dataset with a similar distribution to a target dataset. However, learning domain-invariant representations is challenging due to the large individual differences in EEG signals, which affects the performance of transfer learning methods [100]. In response to this issue, Song et al. proposed a Global Adaptive Transformer for cross-subject enhancement in EEG analysis. The model included a parallel convolutional layer to capture temporal and spatial features and an attention-based adaptor that implicitly transfers source features to target domains, emphasizing the global correlations of EEG features [130].

Task Transfer involves transferring knowledge across different but related tasks. A recent study on multitask learning was proposed as an augmentation strategy to improve performance [131]. Wang et al. proposed a cross-distribution self-supervised pre-training strategy to enhance the model’s generalization ability across multiple datasets. They reported accuracies of 59.2 ± 3.5, 72.6 ± 5.4, 60.2 ± 5.4, and 85.7 ± 2.9 for DEAP, SEED, SEED-IV, and disorders of consciousness (DOC), respectively, supporting the feasibility of using multiple EEG datasets from different tasks to improve the model’s generalization [132]. Furthermore, a ViT model combined with a transfer learning approach for cross-subject emotion recognition achieved an accuracy of 97.55% on the SEED dataset and 88.08% on the SEED-IV dataset [133].

### 5.3. Model Transparency

**Figure 13:**
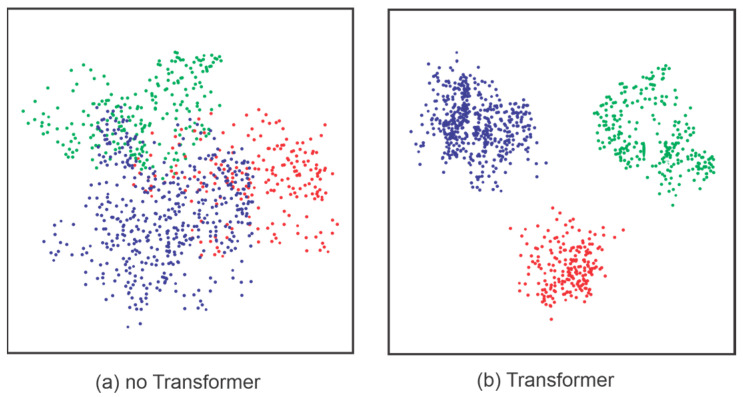
The t-SNE representation of the feature distribution for two experiments, with and without the transformer, clearly shows the separation of features. The different classes have been shown in different colors. This indicates the impact of the attention mechanism in the transformer model.

One of the challenges in modifying transformer models is their limited transparency. The complexity of their multi-layered architecture and large parameter space makes it difficult to interpret how features are processed and prioritized. Several studies have employed methods to track how transformers weigh and prioritize features, including t-distributed Stochastic Neighbor Embedding (t-SNE), visualizing attention weights, and conducting ablation experiments. An ablation experiment involves removing or replacing specific components to assess their contributions to overall model performance [102,103]. t-SNE is an algorithm for dimensionality reduction and visualization, depicted in Figure 13. To demonstrate the capabilities of their model [16], they utilized t-SNE visualization and investigated how it substantially amplifies the discriminative capability of input features.

Visualizing attention weights is another approach to track the processing mechanism of transformers, facilitating further modifications and improvements. For example, Xie et al. benefited from the visualization of attention weights to demonstrate the consistency between the topography of the attention weights and the spectral analysis of the EEG rhythm during the task [134]. Similarly, to reveal the regions of the signal that contribute more to the epileptic labels, aligning more with human expert recognition, ref. [127] visualized the attention weights. They investigated the attention maps from four attention heads and the corresponding EEG signals, claiming that different attention heads, learned in parallel, focus on patterns across different rhythms.

A detailed comparison of studies conducted in recent years is summarized and presented in Table 2.

**Table 2:** Comparison of transformer-based models in EEG analysis in 2024 and 2025.

Study	Dataset	Transformer Model	Application	Performance (ACC%)
[87]	BCI IV-2a BCI IV-2b	Time Series	MI Classification	BCI IV-2a = 88.75%
				BCI IV-2b = 84.20%
[84]	Study Dataset	Time Series	MI Classification	95.24%
[48]	BCI IV-2a BCI IV-2b	Time Series	MI Classification	BCI IV-2a = 77.39%
				BCI IV-2b = 78.20%
[135]	BCI IV-2a BCI IV-2b	Time Series	MI Classification	Model 1:
				BCI IV-2a = 68.75%
				BCI IV-2b = 67.70%
				Model 2:
				BCI IV-2a = 66.55%
				BCI IV-2b = 70.12%
[88]	BCI IV-2a BCI IV-2b	Hybrid	MI Classification	BCI IV-2a = 88.5%
				BCI IV-2b = 88.3%
[85]	BCIC IV-2a BCIC II and III MMIDB	Hybrid	MI Classification	BCIC IV-2a = 86.93%
				BCIC II and III = 94.64%
				MMIDB = 93.52%
[16]	BCI IV-2a BCI IV-2b	Hybrid	MI Classification	Subject-Specific:
				BCI IV-2a = 82.52%
				BCI IV-2b = 88.49%
				Cross-Subject:
				BCI IV-2a = 58.64%
				BCI IV-2b = 76.27%
[71]	Physionet	Hybrid	MI Classification	97.43%
[136]	BCIC2a BCIC2b	Hybrid	MI Classification	BCIC2a = 58.41%
				BCIC2b = 76.18%
[98]	DEAP SEED SEED-IV	Time Series	Emotion Recognition	SEED = 77.34%
				DEAP = 58.31%
				SEED-IV: S1 = 75.00%,
				S2 = 65.83%, S3 = 72.22%
[126]	SEED SEED-IV MPED	Time Series	Emotion Recognition	SEED = 92.58%, Std = 6.00,
				SEED-IV = 83.59%, Std = 12.41,
				MPED = 36.53%, Std = 7.10
[94]	DEAP	Time Series	Emotion Recognition	DEAP: 1-s WS
				V = 95.25%, A = 96.28%
				DEAP: 3-s WS
				V = 95.74%, A = 96.13%
[95]	SEED SEED-IV	Time Series	Emotion Recognition	SEED = 98.82%
				SEED-IV = 96.77%
[100]	SEED SEED-IV	ViT	Emotion Recognition	Intra-Subject:
				SEED = 99.81%%, Std = 0.63,
				SEED-IV = 99.66%, Std = 0.7
				Cross-Subject:
				SEED = 79.19%%, Std = 6.86,
				SEED-IV = 62.38%%, Std = 10.05
[92]	DEAP DREAMER	ViT	Emotion Recognition	DEAP: V = 98.89%,
				A = 98.92%
				DREAMER: V = 99.08%,
				A = 99.21%
[68]	SEED SEED-IV	Swin	Emotion Recognition	Subject-dependent:
				SEED = 94.83%, Std = 7.16,
				SEED-IV = 79.45%, Std = 10.86
				Subject-independent:
				SEED = 80.07%, Std = 10.75,
				SEED-IV = 66.72%, Std = 10.19
[97]	DEAP SEED THU-EP	Hybrid	Emotion Recognition	DEAP: V = 66.14 ± 6.11
				A = 67.83 ± 8.07
				SEED = 87.62 ± 5.43
				THU-EP = 55.42 ± 14.85
[99]	DEAP SEED	Hybrid	Emotion Recognition	DEAP: V = 92.44%
				A = 92.85%
				SEED = 98.69%
[80]	DEAP SEED	Hybrid	Emotion Recognition	DEAP: A-V = 95.73%,
				A = 96.95%,
				V = 96.34%
				SEED = 96.67%
[52]	DEAP SEED	Hybrid	Emotion Recognition	DEAP: V = 98.31%,
				A = 98.28%
				SEED = 94.91%
[2]	DEAP SEED SEED-IV	Hybrid	Emotion Recognition	DEAP = 99.66 ± 0.15
				SEED = 98.85 ± 0.81
				SEED-IV = 99.67 ± 0.12
[69]	SEED SEED-IV SEED-V	Graph	Emotion Recognition	SEED = 95.45%, Std = 4.75
				SEED-IV = 88.62%, Std = 8.01
				SEED-V = 82.53%, Std = 5.84
[93]	SEED SEED-IV DREAMER	Graph	Emotion Recognition	Subject-dependent:
				SEED=96.82%, SEED-IV=82.86%
				Subject-independent:
				SEED=89.66%, SEED-IV=75.78%
				DREAMER:
				V=93.92%, A=94.60%, D= 94.75%
[105]	CHB-MIT Bonn EEG	Time Series	Seizure Prediction	CHB-MIT:
				Sensitivity = 98.24%
				Specificity = 97.27%
				Bonn EEG:
				Binary ACC ~ 99%
				Tertiary Upper ACC ~ 98%
[109]	CHB-MIT	Time Series	Seizure Prediction	Cross-Subject = 74.67%
[110]	CHSZ TUSZ	ViT	Seizure Prediction	CHSZ = 0.650 ± 0.071
				TUSZ = 0.746 ± 0.024
[103]	CHB-MIT	ViT	Seizure Prediction	CHB-MIT = 93.65%
[63]	CHB-MIT SH-SDU	ViT	Seizure Prediction	CHB-MIT = 97.57%
				SH-SDU = 95.88%
[102]	CHB-MIT	Hybrid ViT	Seizure Prediction	93.65%
[104]	Turkish Epilepsy EEG Dataset	Hybrid	Seizure Prediction	Turkish Epilepsy
				EEG Dataset = 95.99%
[111]	CHB-MIT	Hybrid	Seizure Prediction	94.1%
[3]	CHB-MIT	Hybrid	Seizure Prediction	Sensitivity = 99.75
[107]	CHB-MIT	Graph	Seizure Prediction	98.71%
[106]	CHB-MIT	Hybrid, Graph, Swin	Seizure Prediction	94.75%

## 6. Discussion

This paper provides a comprehensive overview of transformer architecture, its variants, and their applications in EEG analysis, highlighting key challenges and mitigation strategies for enhancing model performance. Transformer architecture has made a significant impact across various domains, and its application in EEG analysis is rapidly increasing. With the ability to handle both short- and long-range dependencies, along with an end-to-end structure, transformers have shown great potential in improving the performance of EEG models. In this review, we outline transformer architectures such as Time Series, Vision, Graph, and hybrid models, each applied to different characteristics of EEG data. Time series models, by leveraging positional encoding and attention mechanisms, have shown success in capturing long-range dependencies in EEG data, particularly in motor imagery studies. Vision models, where EEG data are transformed into images, capture the spatial and spectral features in EEG data and are primarily used in seizure prediction studies. Graph Attention models, where EEG data are represented as a connectivity graph, capture spatial and spectral dependencies, as well as interactions between different regions of the brain, and are applied across a wide range of EEG studies. Hybrid models combine the strengths of CNNs for local feature extraction with transformers for global dependency modeling, making them ideal to capture the spatial, temporal, and spectral features present in EEG signals, and are mostly used in emotion classification tasks.

This review categorizes the most frequent transformer applications in EEG analysis into motor imagery classification, emotion classification, and seizure detection. This also illustrates how the public availability of datasets influences the frequency of certain studies, confirming that a major issue reported in the reviewed studies is data scarcity. Two prominent strategies to mitigate the challenges of limited data and model generalization are data augmentation and transfer learning. We review some successful data augmentation studies aimed at increasing the size and diversity of training datasets, thereby preventing overfitting and improving model generalization. While data augmentation has been shown to enhance model performance in some studies, its impact can be incremental, suggesting the need for more sophisticated approaches in future work. Additionally, some studies have utilized transfer learning strategies to overcome the challenge of limited labeled data. By leveraging knowledge from pre-trained models on large, domain-relevant datasets, transformers can be fine-tuned for specific EEG tasks. The adaptability of transfer learning offers a significant opportunity for future studies to improve model performance, particularly when working with small or diverse EEG datasets.

## Figures and Tables

**Table 1:** Summary of dataset abbreviations and their corresponding full names reviewed in this study.

Dataset Abbreviation	Full Dataset Name
BCI IV-2a	BCI Competition IV Dataset 2a
BCI IV-2b	BCI Competition IV Dataset 2b
BCIC II and III	BCI Competition II and III
Bonn EEG	Bonn EEG Dataset
CHB-MIT	Children’s Hospital Boston MIT EEG
CHSZ	Chinese Epilepsy EEG Dataset
DEAP	Database for Emotion Analysis using Physiological Signals
DREAMER	Dataset for Emotion Recognition using EEG and Motion
MMIDB	Multi-Modal Individual Dataset Database
SEED	Sensing Emotion and Emotion Dataset
SEED-IV	Sensing Emotion and Emotion Dataset IV
SEED-V	Sensing Emotion and Emotion Dataset V
SH-SDU	Shenzhen University EEG Dataset
THU-EP	Tsinghua University Epileptic EEG Dataset
TUSZ	Tsinghua University Sleep Dataset

## Nomenclature

The following abbreviations are used in this manuscript:

A	Arousal
ACC	Accuracy
BCI	Brain–Computer Interface
CSP	Common Spatial Patterns
CNN	Convolutional Neural Network
CV	Cross-Validation
DA	Data Augmentation
DE	Differential Entropy
DOC	Disorders of Consciousness
EEG	Electroencephalography
EOG	Electrooculography
EMG	Electromyography
FFN	Feed-Forward Network
GAT	Graph Attention Transformer
GAN	Generative Adversarial Network
GCN	Graph Convolutional Network
KNN	K-nearest neighbors
LM	Language Model
LOSO CV	Leave-One-Subject-Out Cross-Validation
LSTM	Long Short-Term Memory
MI	Motor Imagery
MLP	Multilayer Perceptron
NLP	Natural Language Processing
PCC	Pearson Correlation Coefficients
PSD	Power Spectral Density
ReLU	Rectified Linear Unit
RGB	Red, Green, Blue
RSVP	Rapid Serial Visual Presentation
SSVEPs	Steady-State Visual Evoked Potentials
Std	Standard Deviation
STFT	Short-Time Fourier Transform
SVM	Support Vector Machine
t-SNE	t-distributed Stochastic Neighbor Embedding
V	Valence
ViT	Vision Transformer
WS	Window Size
